# The Effects of L-Citrulline on the Apparent Digestion and Metabolism of Nutrients, Blood Hormone Levels, Amino Acid Metabolism and the Diversity of Faecal Microbiota in Mares in the Later Stage of Pregnancy

**DOI:** 10.3390/life16050744

**Published:** 2026-04-30

**Authors:** Pengshun Liu, Fan Yang, Jiahao Li, Chao Li, Xinsheng Guo, Xiaobin Li

**Affiliations:** 1 Xinjiang Herbivore Nutrition Laboratory for Meat & Milk, College of Animal Science, Xinjiang Agricultural University, Urumqi 830052, China; m18439437962@163.com (P.L.); yangfan312au@163.com (F.Y.); xjauljh@163.com (J.L.); 18324052590@163.com (C.L.); 13017602713@163.com (X.G.); 2 Xinjiang Key Laboratory of Horse Breeding and Exercise Physiology, College of Animal Science, Xinjiang Agricultural University, Urumqi 830052, China

**Keywords:** L-citrulline, late-pregnant mares, digestibility and metabolism, hormones, fecal microbiota

## Abstract

This study aimed to investigate the impact of dietary L-citrulline supplementation on the health of mares during late gestation. Thirty-two healthy mares in late pregnancy were randomly assigned to four groups: a control group (CON, 0 g/d) and three treatment groups receiving 15, 30, and 45 g/d/head of L-citrulline, respectively. The trial spanned 72 days, including a 12-day adaptation phase and a 60-day formal feeding period. A fixed daily feeding amount of 11.2 kg/head was provided, ensuring complete consumption and consistent dry matter intake across all groups. Results demonstrated that supplementation with 30 g/d/head of L-citrulline significantly improved the apparent digestibility of crude protein and nitrogen metabolism rate (*p* < 0.05), while notably increasing plasma superoxide dismutase (SOD) activity (*p* < 0.01) and reducing plasma malondialdehyde (MDA) concentration by 10.53% (*p* < 0.01). Furthermore, mares receiving 30 g/d of L-citrulline showed a 14.81% increase in plasma estradiol (E2) concentration (*p* < 0.01). Urinary concentrations of E2, estrone sulfate (ESS), and 17α-dihydroequilin sulfate (17α-DHEQS) were also significantly elevated (*p* < 0.05). This supplementation also enhanced plasma amino acid levels related to the urea cycle and improved the diversity of fecal microbiota, increasing the abundance of beneficial bacteria. A multi-indicator scoring system identified 30 g/d as the optimal supplemental dose of L-citrulline. These findings suggest that 30 g/d of L-citrulline may act as a nutritional regulator, offering valuable insights for enhancing the physiological and metabolic health of mares during late gestation.

## 1. Introduction

The late gestation period is a critical phase during which mares experience considerable metabolic and physiological stress. The rapid growth of the fetus, which accounts for two-thirds of its final body weight, significantly increases the nutritional demands and metabolic load on the dam, often accompanied by health risks such as heightened oxidative stress, altered immune regulation, and disturbances in the gut microbiota [1,2]. Therefore, nutritional interventions to support metabolic health, hormonal balance, and intestinal homeostasis during this stage are essential for optimal fetal development and maternal well-being. Arginine, a functional amino acid, plays a pivotal role in enhancing vascular function, improving antioxidant capacity, modulating immunity, and promoting protein synthesis [3]. Research in ruminants and pigs has demonstrated that arginine supplementation can improve placental blood flow and reproductive performance via the nitric oxide (NO) pathway [4,5]. However, arginine experiences a significant “first-pass effect” in the gut and liver, limiting its oral bioavailability [6]. L-citrulline, as the direct precursor of arginine, bypasses this first-pass effect through its unique metabolic pathway. It efficiently and steadily increases circulating arginine levels in vivo, making it a promising functional nutrient [7,8,9]. The beneficial effects of L-citrulline have been widely reported in monogastric animals such as mice and pigs, including enhanced antioxidant function and improved vascular endothelial health [10,11]. Positive impacts on reproductive performance have also been noted in ruminants like sheep [12]. However, research on L-citrulline supplementation in equines, particularly during pregnancy, remains limited. The optimal supplementation dose and its dose-dependent effects on various physiological functions in pregnant mares have not been fully elucidated. To address this gap, this study investigated the effects of L-citrulline supplementation in late-pregnant Yili mares. A dose-dependent approach was used to evaluate the impact of varying doses on nutrient digestibility and metabolism, blood biochemical and antioxidant indices, reproductive hormone levels, plasma amino acid metabolism, and fecal microbiota diversity. Additionally, a multi-indicator scoring system was employed to identify the optimal supplementation dose. This study aims to provide a scientific foundation for developing nutritional strategies to support the health of pregnant mares.

## 2. Materials and Methods

### 2.1. Animals and Experimental Design

The experimental protocol was approved by the Animal Care and Use Committee of Xinjiang Agricultural University (Urumqi, China) (Approval No. 2022020/2022-05). The study was conducted at Zhaosu Horse Farm in Zhaosu County, Ili Kazakh Autonomous Prefecture, Xinjiang, China (43°09′ to 43°15′ N, 80°08′ to 81°30′ E). All procedures adhered to relevant guidelines and regulations for animal experimentation. L-Citrulline (L-Cit, purity ≥ 99% on a dry matter [DM] basis) was sourced from Anhui Runtai Biotechnology Co., Ltd. (Hefei, Anhui, China).

Thirty-two healthy, late-pregnant (8–9 months of gestation) Yili mares, with similar expected parturition dates (March-April), parity (6–7), age (9–10 years), and body weight (455.1 ± 8.8 kg), were selected for the study. Using a computer-generated random number sequence, the mares were randomly assigned to four groups (*n* = 8 per group): a control group (CON) and three treatment groups (Test I, II, and III). The CON group was fed only the basal diet, while the treatment groups received the basal diet supplemented with 15 (Test I), 30 (Test II), and 45 (Test III) g/d/head of L-citrulline, respectively. The trial spanned 72 days, comprising a 12-day adaptation period followed by a 60-day experimental period. The supplementation of L-citrulline began at the start of the 9th month of gestation and ended at the conclusion of the 10th month. All mares were maintained under identical management and nutritional conditions. The selected L-citrulline doses were based on previous related studies [13,14].

The sample size of 8 mares per group (*n* = 8) was determined based on common practices in similar nutritional intervention studies [13,15]. Given the availability of experimental animals and resource constraints, this sample size was considered sufficient for preliminary evaluation in this exploratory dose–response study.

All mares were individually housed in single stalls. The total mixed ration (TMR) was provided four times daily at 10:00, 14:00, 18:00, and 22:00, with each feeding comprising 2.80 kg, totaling 11.20 kg per day. The daily dose of L-citrulline for each group was accurately weighed using an electronic balance. At 10:00 each day, during the first feeding, the weighed L-citrulline was thoroughly mixed with the TMR and provided to each mare. Feed consumption was monitored to ensure complete intake. Stalls were cleaned immediately after each feeding, and clean drinking water was available at all times. Regular disinfection of the stalls was carried out throughout the trial period. The composition and nutritional levels of the TMR diet are detailed in Table 1.

### 2.2. Sample Collection and Processing

During the formal experimental period, blood samples were collected from the jugular vein of all mares before the morning feeding on days 0, 30, and 60. The blood was drawn into heparinized tubes, centrifuged, and the plasma was separated. The plasma was aliquoted and stored at −20 °C for subsequent analysis of blood biochemical indices, antioxidant status, hormone levels, and amino acid metabolomics (using samples from day 60). Simultaneously, 24 h urine samples were collected from each mare, aliquoted, and stored under the same conditions for the measurement of urinary estrogen concentrations.

Two digestion and metabolism trials were conducted during the formal experimental period, from days 26–30 and 56–60 (5 days per trial). A total collection method for feces and urine was employed. The total daily fecal output from each mare was thoroughly mixed, and a 10% aliquot of the total fresh weight was taken. This aliquot was then divided into two portions. One portion (10% of the 10% aliquot) was treated with a 10% sulfuric acid solution (10 mL per 100 g of fecal sample), thoroughly mixed, and stored at −20 °C for subsequent crude protein (CP) analysis. The other portion (90% of the 10% aliquot) was dried in a 65 °C oven, ground through a 40-mesh sieve, thoroughly mixed, and stored at −20 °C for the determination of other nutrients. For urine collection, a field-adapted equine urine collection device was used. The total daily urine output from each mare was thoroughly homogenized in a collection bucket. A 10% sulfuric acid solution was then added to the bucket to prevent nitrogen loss. The urine was filtered through four layers of gauze, and a 10% aliquot of the total volume was collected into sampling tubes and stored at −20 °C.

### 2.3. Determination of Indicators and Methods

Parameters measured in the collected feed, fecal, and urine samples included DM, organic matter (OM), gross energy (GE), CP, neutral detergent fiber (NDF), acid detergent fiber (ADF), calcium (Ca), and phosphorus (P) contents. Determinations were conducted according to the relevant Chinese National Standards: GB/T 6435-2014 (DM), GB/T 6438-2007 (OM), GB/T 14489.1-2008 (GE), GB/T 6432-2018 (CP), NY/T 1459-2007 (NDF), NY/T 1393-2007 (ADF), GB/T 6436-2018 (Ca), and GB/T 6437-2023 (P). The apparent digestibility of nutrients was calculated using the total fecal collection method with the following formula: Apparent digestibility of nutrient (%) = [(Nutrient intake − Fecal nutrient output)/Nutrient intake] × 100%. The apparent metabolic rate was calculated as: [(Nutrient intake − Fecal nutrient output − Urinary nutrient output)/Nutrient intake] × 100%.

Blood biochemical indices were analyzed using an automated biochemical analyzer, which included: total protein (TP), albumin (ALB), globulin (GLB), creatinine (CREA), urea (UREA), glucose (Glu), total bilirubin (T-BiL), total cholesterol (TC), alanine aminotransferase (ALT), aspartate aminotransferase (AST), alkaline phosphatase (ALP), gamma-glutamyl transferase (γ-GT), creatine kinase (CK), lactate dehydrogenase (LDH), calcium (Ca^2+^), inorganic phosphorus (Inorganic P), and magnesium (Mg^2+^). These were measured using a Mindray Bio-Medical Electronics Co., Ltd. automated biochemical analyzer (model BS-240VET, Shenzhen, China) with commercial reagent kits (Mindray Bio-Medical Electronics Co., Ltd., Shenzhen, China) following the manufacturer’s protocols.

Plasma antioxidant indices, including total antioxidant capacity (T-AOC), superoxide dismutase (SOD), glutathione peroxidase (GSH-Px), catalase (CAT), and malondialdehyde (MDA). were analyzed using a UV-Vis spectrophotometer (Agilent Technologies, Santa Clara, CA, USA, Model: Cary 60) according to the manufacturer’s instructions provided with the assay kits, which were supplied by Beijing Hua Ying Bioengineering Institute (Beijing, China).

Plasma hormone levels, including testosterone (T), progesterone (P4), estradiol (E2), follicle-stimulating hormone (FSH), luteinizing hormone (LH), and gonadotropin-releasing hormone (GnRH), were measured, along with urinary hormone levels, including estrone (E1) and estradiol (E2). These plasma and urinary hormone levels were determined using a microplate reader (Agilent Technologies, Model: BioTek 800 TS) following the manufacturer’s instructions provided with the enzyme-linked immunosorbent assay (ELISA) kits, which were supplied by Beijing Hua Ying Bioengineering Institute (Beijing, China).

Urinary conjugated estrogen indices, including sodium estrone sulfate (ESS), sodium equilin sulfate (EQS), and sodium 17α-dihydroequilin sulfate (17α-DHEQS), were determined using Ultra-High Performance Liquid Chromatography (HPLC), following the method described by Yao [16].

The plasma amino acid profile was analyzed using high-performance liquid chromatography-tandem mass spectrometry (HPLC-MS/MS). Chromatographic separation was performed on a C18 reversed-phase column (4.6 × 150 mm, 5 μm) with a mobile phase consisting of acetonitrile-water (containing 0.1% formic acid and 0.01% heptafluorobutyric acid) under a gradient elution program at a flow rate of 1.0 mL/min. Mass spectrometric detection was conducted using an electrospray ionization (ESI) source in positive ion mode with multiple reaction monitoring (MRM). The concentration of each amino acid was quantified using its corresponding standard calibration curve.

Fresh fecal samples were collected via rectal sampling on the final day of the experiment (day 60). For pH measurement, 10 g of fresh feces was placed into a clean tube, diluted with 10 mL of distilled water (feces-to-water ratio 1:1), and thoroughly homogenized to dissolve pH-active substances. A calibrated pH meter probe was inserted into the fecal suspension, gently stirred, and the reading was recorded after stabilization. The concentration of fecal short-chain fatty acids (SCFAs) was determined by gas chromatography (GC) at Novogene Co., Ltd. (Beijing, China). DNA extraction from fecal samples, 16S rRNA gene amplification, and high-throughput sequencing were performed by Novogene Co., Ltd. (Beijing, China). Subsequent bioinformatics analysis was conducted on their online platform, with data analysis methods detailed in Section 2.4. Correlation analysis was performed using Pearson’s correlation method.

### 2.4. Data Processing and Statistical Analysis

All data are presented as the mean ± standard error of the mean (SEM). Statistical analysis was conducted using SPSS 26.0 software, with a significance level set at *p* < 0.05. Data distribution normality and homogeneity of variances were assessed initially. For data measured at multiple time points (nutrient digestibility and metabolism rates, blood biochemical parameters, antioxidant capacity, and hormone levels), repeated measures analysis of variance (ANOVA) was used, with “treatment group (TRT)” as the between-subject factor and “sampling time (Date)” as the within-subject factor. For data measured only at the end of the trial, one-way ANOVA was used to compare groups. When parametric test assumptions were not met, non-parametric tests were applied: the Friedman test for repeated measures data and the Kruskal–Wallis test for independent samples. Multiple comparisons following parametric tests were conducted using Duncan’s method. Plasma amino acid data were processed using principal component analysis (PCA) and orthogonal partial least squares-discriminant analysis (OPLS-DA) to identify differential amino acids with a variable importance in projection (VIP) score greater than 1 and a significance level of *p* < 0.05. Pearson’s correlation analysis was used to assess associations between variables. For 16S rRNA gene sequencing data, bioinformatic analysis was conducted on the NovoMagic online cloud platform (https://magic.novogene.com), with differential taxa significance *p*-values subjected to false discovery rate (FDR) correction

To determine the optimal dose of L-citrulline, this study employed a composite multi-indicator scoring method. This approach aligns with the integrated assessment framework used in equine nutritional performance evaluation [17] and adheres to the principles of standardized scoring systems applied in equine health science [18]. Specific procedures included: (1) screening key indicators showing significant intergroup differences (*p* < 0.05) within each module; (2) standardizing each indicator to a 0–100 scale according to preset benefit/cost directionality, adopting an unweighted aggregation scheme consistent with multi-indicator evaluation optimization concepts [19]; (3) summing the standardized values to calculate individual composite health scores; and (4) comparing group scores via one-way analysis of variance (ANOVA).

## 3. Results

### 3.1. Effects of Supplementing L-Citrulline on the Apparent Digestion and Metabolism of Nutrients in Pregnant Mares

Repeated measures ANOVA was conducted to assess the impact of L-citrulline supplementation on nutrient digestibility and metabolism in pregnant mares (Table 2). The treatment main effect analysis revealed that, compared to the CON group, supplementation with 30 g/d of L-citrulline (Test II group) significantly improved the apparent digestibility of CP, NDF, and ADF (*p* < 0.05), and increased the nitrogen metabolism rate by 19.97% (η^2^ = 0.38; *p* < 0.01). No significant differences were observed among groups for DM digestibility, OM digestibility, phosphorus digestibility, phosphorus metabolic rate, digestible energy, or metabolizable energy (*p* > 0.05). However, the pattern of change in nitrogen metabolic rate showed that the benefit of supplementation peaked at 30 g/d and declined to levels comparable to the control group at 45 g/d, suggesting that the effect of L-citrulline may occur within an optimal dose window rather than follow a simple linear relationship.

The time main effect analysis showed that sampling time significantly influenced NDF digestibility, ADF digestibility, metabolizable energy, and phosphorus metabolic rate (*p* < 0.05), and had a highly significant effect on digestible energy and calcium metabolic rate (*p* < 0.01). The interaction between treatment and time was not significant for any of the observed digestibility and metabolism parameters (*p* > 0.05).

**Table 2:** Effects of Supplementing L-citrulline on the apparent digestion and metabolism of nutrients in pregnant mares.

Items	Groups				SEM	*p*-Value		
	CON	Test I	Test II	Test III		TRT	Date	TRT*Date
Dry matter digestibility %	55.23	55.13	57.76	56.19	1.25	0.42	0.49	0.95
Organic matter digestibility %	61.01	61.69	61.61	59.75	1.11	0.58	0.95	0.20
Digestive energy MJ/kg	8.91	8.95	9.38	8.80	0.21	0.22	0.00	0.47
Crude protein digestibility %	64.98 ^ABb^	65.56 ^ABb^	70.27 ^Aa^	63.51 ^Bb^	1.41	0.01	0.41	0.50
NDF digestibility %	40.21 ^b^	42.03 ^ab^	45.73 ^a^	40.85 ^ab^	1.70	0.11	0.04	0.70
ADF digestibility %	35.49 ^b^	36.40 ^ab^	42.64 ^a^	36.78 ^ab^	2.21	0.10	0.01	0.84
Ca digestibility %	44.16 ^a^	44.25 ^ab^	47.60 ^ab^	42.23 ^b^	1.63	0.14	0.00	0.38
P digestibility %	43.68	38.47	42.67	43.65	2.09	0.25	0.34	0.22
Metabolic energy MJ/kg	7.97	7.96	8.45	7.79	0.22	0.20	0.02	0.61
N metabolic rate%	27.26 ^Bb^	28.45 ^ABb^	35.43 ^Aa^	25.44 ^Bb^	2.05	0.01	0.28	0.97
Ca metabolic rate %	26.65 ^ab^	24.15 ^ab^	29.98 ^a^	23.05 ^b^	1.97	0.07	0.00	0.31
P metabolic rate %	25.22	22.45	25.45	24.50	1.96	0.69	0.01	0.69

Note: Within a row, means with different lowercase superscript letters (a, b) differ significantly at *p *< 0.05, and with different uppercase superscript letters (A, B) differ highly significantly at *p *< 0.01. The same superscript notation applies to Table 3, Table 4 and Table 5.

### 3.2. The Effect of Supplementing L-Citrulline on the Blood Biochemical Indicators of Pregnant Mares

The effects of L-citrulline on blood biochemical indices were evaluated using repeated measures ANOVA (Table 3). The treatment main effect analysis indicated that, compared to the CON group, the Test II group (30 g/d L-citrulline) significantly reduced the concentration of T-BiL by 25.38% (*p* < 0.05) and the activity of AST by 9.96% (*p* < 0.05). Additionally, the blood CREA concentration in the Test II group was significantly lower than in all other groups, including the CON group, showing a 9.77% reduction (*p* < 0.05). However, blood ALP activity in the Test II group was significantly higher than in the CON group, with an increase of 22.47% (*p* < 0.01). Time analysis revealed significant effects of sampling time on the concentrations of TP, GLB, CREA, LDH, and Mg^2+^ (*p* < 0.05), and highly significant effects on T-BiL, Glu, ALP, CK, Ca^2+^, and P (*p* < 0.01).

**Table 3:** Effects of Supplementing L-citrulline on blood Biochemical indicators of pregnant mares.

Items	Groups				SEM	*p*-Value		
	CON	Test I	Test II	Test III		TRT	Date	TRT*Date
TP (g/L)	72.75	72.51	69.54	72.40	1.08	0.13	0.01	0.49
ALB (g/L)	30.97	30.32	29.52	30.11	0.49	0.22	0.58	0.25
GLB (g/L)	41.78	42.19	40.02	42.29	1.19	0.51	0.02	0.71
UREA (mmol/L)	5.63	5.68	6.07	5.92	0.28	0.66	0.27	0.99
CREA (mmol/L)	117.95 ^ABab^	111.84 ^Bb^	106.43 ^Bc^	124.62 ^Aa^	3.15	0.00	0.01	0.43
TC (mmol/L)	2.53	2.49	2.40	2.55	0.08	0.56	0.15	0.91
T-BiL (µmol/L)	21.71 ^a^	18.21 ^ab^	16.20 ^b^	21.59 ^a^	1.56	0.04	0.00	0.96
Glu (mmol/L)	4.90	4.83	4.61	4.59	0.13	0.22	0.00	0.07
ALT (U/L)	4.99	4.86	4.12	4.76	0.30	0.18	0.52	0.75
AST (U/L)	328.60 ^a^	317.66 ^ab^	295.88 ^b^	315.39 ^a^	9.07	0.09	0.76	0.77
ALP (U/L)	161.44 ^Bb^	179.87 ^ABab^	197.71 ^Aa^	177.81 ^ABab^	8.20	0.03	0.00	0.63
γ-GT (U/L)	13.42	13.91	13.67	13.31	0.72	0.94	0.89	0.87
CK (U/L)	340.62	283.83	336.18	283.23	19.82	0.06	0.00	0.94
LDH (U/L)	430.54	434.23	447.28	445.44	19.00	0.90	0.04	0.83
AST/ALT	69.89	71.48	77.72	67.58	5.07	0.54	0.32	0.83
Ca^2+^ (mmol/L)	3.03 ^a^	2.98 ^ab^	2.96 ^ab^	2.95 ^b^	0.03	0.15	0.00	0.10
Mg^2+^ (mmol/L)	0.90	1.04	1.01	0.91	0.09	0.61	0.01	0.93
P (mmol/L)	1.09	1.07	1.10	1.11	0.05	0.93	0.00	0.59

### 3.3. The Effect of Supplementing L-Citrulline on the Antioxidant Capacity of Plasma in Pregnant Mares

The effects of L-citrulline supplementation on the plasma antioxidant status of mares were assessed using repeated measures ANOVA, as shown in Table 4. The treatment main effect analysis revealed that L-citrulline supplementation significantly enhanced antioxidant function in the mares. Compared to the CON group, the Test II group (30 g/d L-citrulline) significantly increased plasma SOD activity by 10.90% (*p* < 0.01) and significantly decreased plasma MDA concentration by 11.53% (η^2^ = 0.27; *p* < 0.01). The Test III group (45 g/d L-citrulline) also significantly increased plasma T-AOC by 15.80% compared to the CON group (*p* < 0.01). The time main effect analysis indicated that sampling time had a highly significant effect on the plasma concentrations of SOD, CAT, GSH-Px, T-AOC, and MDA (*p* < 0.01). The interaction between treatment and time was significant only for CAT activity (*p* < 0.05) and not for the other antioxidant indices.

**Table 4:** Effects of Supplementing L-citrulline on the antioxidant capacity of plasma in pregnant mares.

Items	Groups				SEM	*p*-Value		
	CON	Test I	Test II	Test III		TRT	Date	TRT*Date
SOD (U/mL)	77.74 ^Bb^	84.19 ^ABa^	86.21 ^Aa^	82.41 ^ABab^	2.03	0.03	0.00	0.18
CAT (U/mL)	33.66 ^ab^	33.47 ^b^	36.96 ^a^	34.04 ^ab^	1.12	0.10	0.00	0.03
GSH-Px (U/mL)	136.00	140.62	139.91	140.15	1.96	0.32	0.00	0.18
T-AOC (U/mL)	8.61 ^Bb^	9.19 ^ABab^	9.42 ^ABab^	9.97 ^Aa^	0.28	0.01	0.00	0.32
MDA (nmol/mL)	3.73 ^Aa^	3.42 ^ABb^	3.30 ^Bb^	3.37 ^ABb^	0.11	0.03	0.00	0.34

### 3.4. The Effect of Supplementing L-Citrulline on Hormone Concentrations in Plasma and Urine of Pregnant Mares

The effects of L-citrulline on reproductive hormones were evaluated using repeated measures ANOVA, as shown in Table 5. The treatment main effect analysis revealed that, compared to the CON group, the Test II group (30 g/d L-citrulline) significantly increased plasma E2 concentration by 14.81% (η^2^ = 0.33; *p* < 0.01). In urine, the concentrations of ESS, 17α-DHEQS, and E2 in the Test II group were also significantly higher than in the CON group, with increases of 8.05%, 11.71%, and 12.16%, respectively (*p* < 0.05). The time main effect analysis indicated that sampling time had a highly significant effect on plasma concentrations of GnRH, LH, FSH, and P4, as well as on urinary concentrations of ESS, 17α-DHEQS, E1, and E2 (*p* < 0.01).

**Table 5:** Effects of Supplementing L-citrulline on hormone concentrations in plasma and urine of pregnant mares.

Items		Groups				SEM	*p*-Value		
		CON	Test I	Test II	Test III		TRT	Date	TRT*Date
Plasma	GnRH (pg/mL)	43.78	43.24	41.85	42.47	0.89	0.44	0.00	0.87
	LH (mIU/mL)	8.15	8.16	8.19	8.24	0.32	1.00	0.00	1.00
	FSH (mIU/mL)	3.63	3.51	3.75	3.75	0.18	0.73	0.00	0.16
	E2 (pg/mL)	32.27 ^Bb^	33.24 ^ABb^	37.05 ^Aa^	32.68 ^ABb^	1.15	0.02	0.55	0.21
	P4 (ng/mL)	120.96	129.51	136.97	119.06	7.53	0.31	0.00	0.34
	T (ng/mL)	0.75	0.44	0.46	0.53	0.11	0.15	0.65	0.16
Urine	E1 (pg/mL)	26.06	27.13	27.43	26.50	0.77	0.59	0.00	0.82
	E2 (pg/mL)	3.29 ^b^	3.71 ^a^	3.69 ^a^	3.54 ^ab^	0.12	0.05	0.00	0.84
	ESS (μg/mL)	95.96 ^b^	101.35 ^ab^	103.68 ^a^	101.82 ^a^	1.95	0.04	0.00	0.53
	EQS (μg/mL)	48.92	52.98	53.17	50.97	1.75	0.28	0.03	0.91
	17α-DHEQS (μg/mL)	19.22 ^b^	20.25 ^ab^	21.47 ^a^	20.43 ^ab^	0.62	0.09	0.00	0.13

### 3.5. Optimal L-Citrulline Dose Determined by Multi-Indicator Scoring

**Figure 1:**
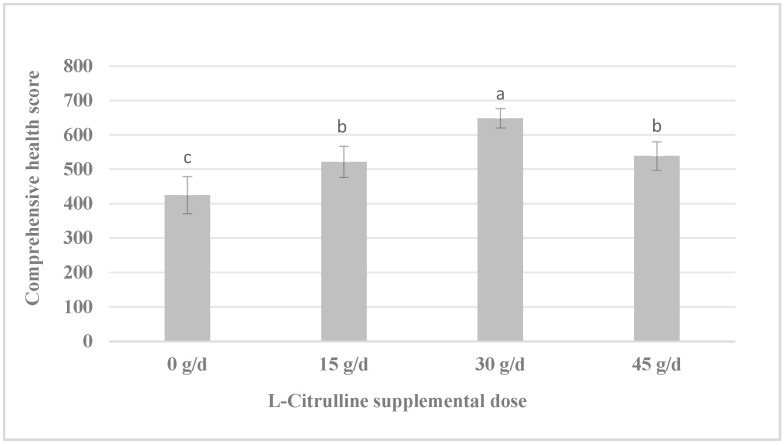
Comprehensive health score of mares supplemented with L-citrulline. Different lowercase letters (a, b, c) above the bars indicate significant differences among groups (*p* < 0.05, Duncan’s multiple range test). CON: 0 g/d; Test I: 15 g/d; Test II: 30 g/d; Test III: 45 g/d.

To quantitatively assess and compare the overall effects of different L-citrulline doses, a comprehensive health score was calculated for each experimental mare based on key indicators that showed significant inter-group differences in Section 3.1, Section 3.2, Section 3.3 and Section 3.4. One-way ANOVA revealed a significant difference in the comprehensive health scores among the different treatment groups (*p* < 0.01, Figure 1). Duncan’s multiple range test indicated that the comprehensive score of the Test II group (supplemented with 30 g/d L-citrulline) was significantly higher than those of the CON group, the 15 g/d group (Test I), and the 45 g/d group (Test III) (*p* < 0.05). No significant differences were found among the latter three groups (*p* > 0.05). These results demonstrate that, within the dose range evaluated in this study, the supplementation of 30 g/d L-citrulline achieved the highest comprehensive health score.

### 3.6. The Effect of Supplementing L-Citrulline on Plasma Amino Acid Metabolism in Pregnant Mares

To investigate the long-term effects of L-citrulline supplementation on amino acid metabolism, targeted amino acid metabolomics analysis was performed on plasma samples collected on day 60 from the control (CON) and 30 g/d L-citrulline (optimal dose, Test II) groups.

**Figure 2:**
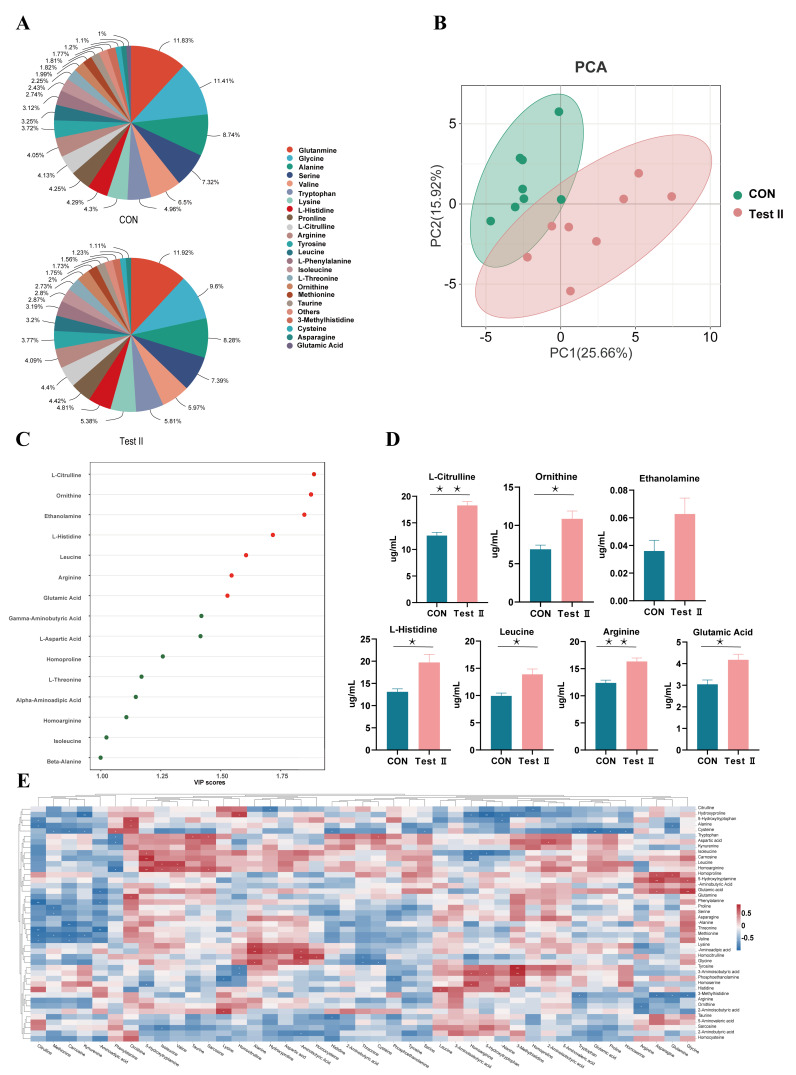
Effects of L-citrulline supplementation on plasma amino acid metabolism in pregnant mares. (**A**) Relative percentage composition of plasma amino acids. (**B**) Principal component analysis (PCA) score plot based on all targeted and quantified amino acids. (**C**) Variable importance in projection (VIP) scores obtained from the orthogonal partial least squares-discriminant analysis (OPLS-DA) model, showing the top 15 differential amino acids (VIP > 1.0 and *p* < 0.05). (**D**) Absolute concentrations of 7 key amino acids with VIP > 1.5. Inter-group differences were compared by one-way ANOVA. Data are presented as mean ± SEM. * *p* < 0.05, ** *p* < 0.01. (**E**) Pearson correlation heatmap of plasma amino acids between the CON and Test II groups. The color of each square represents the magnitude and direction of the correlation coefficient (r); red indicates a positive correlation, blue indicates a negative correlation, and a darker color represents a stronger correlation (* *p* < 0.05, ** *p* < 0.01, *** *p* < 0.001).

Analysis of plasma amino acid composition (Figure 2A) revealed that 23 amino acids with a relative abundance >1% were detected in both groups. Glutamine, glycine, alanine, serine, and valine were the most abundant (each > 5%). Compared to the CON group, the Test II group exhibited an increased proportion of amino acids associated with the urea cycle, including citrulline, arginine, and ornithine. PCA showed a separation trend of the CON and Test II group sample points along the first principal component (PC1) in the PCA score plot (Figure 2B), suggesting a notable difference in the amino acid metabolic profiles between the two groups. To identify key amino acids responsible for the inter-group differences, OPLS-DA was performed. This OPLS-DA model underwent 7-fold cross-validation, with model parameters of R^2^Y = 0.895 and Q^2^ = 0.673. The permutation test *p*-value for Q^2^ was less than 0.01, confirming the model’s stability, reliability, and predictive capability without overfitting. Based on this model, VIP values were determined. Using the criteria of VIP > 1.0 and *p* < 0.05, 15 differential amino acids were identified, including 4 essential and 11 non-essential amino acids (Figure 2C). Among these, citrulline (VIP = 1.89), ornithine, histidine, leucine, arginine, and glutamate had VIP scores greater than 1.5. Quantitative analysis of these 7 key amino acids with VIP > 1.5 showed that, compared to the CON group, the Test II group exhibited significantly higher plasma concentrations of citrulline and arginine (*p* < 0.01), along with significantly increased concentrations of ornithine, histidine, leucine, and glutamate (*p* < 0.05) (Figure 2D). Correlation heatmap analysis (Figure 2E) revealed that L-citrulline supplementation altered the correlation network structure among plasma amino acids. Specifically, in the Test II group, citrulline and ornithine showed a strong negative correlation (*p* < 0.01), while citrulline exhibited only a weak positive correlation with its direct metabolic product, arginine (*p* < 0.05). This altered correlation pattern suggests that L-citrulline supplementation may influence the metabolic balance of intermediates in the urea cycle.

### 3.7. The Effect of Supplementing L-Citrulline on the VFA Concentration and Flora Diversity in the Faeces of Pregnant Mares

#### 3.7.1. The Effect of Supplementing L-Citrulline on the pH and VFA of Faeces in Pregnant Mares

As shown in Table 6, no significant differences (*p* > 0.05) were observed between the mares in the Test II group (supplemented with 30 g/d L-citrulline) and the CON group in terms of fecal pH or the concentrations of acetate, propionate, butyrate, isobutyrate, valerate, isovalerate, and total volatile fatty acids.

#### 3.7.2. The Effect of Supplementing L-Citrulline on the Diversity of Faecal Microbiota in Pregnant Mares

**Figure 3:**
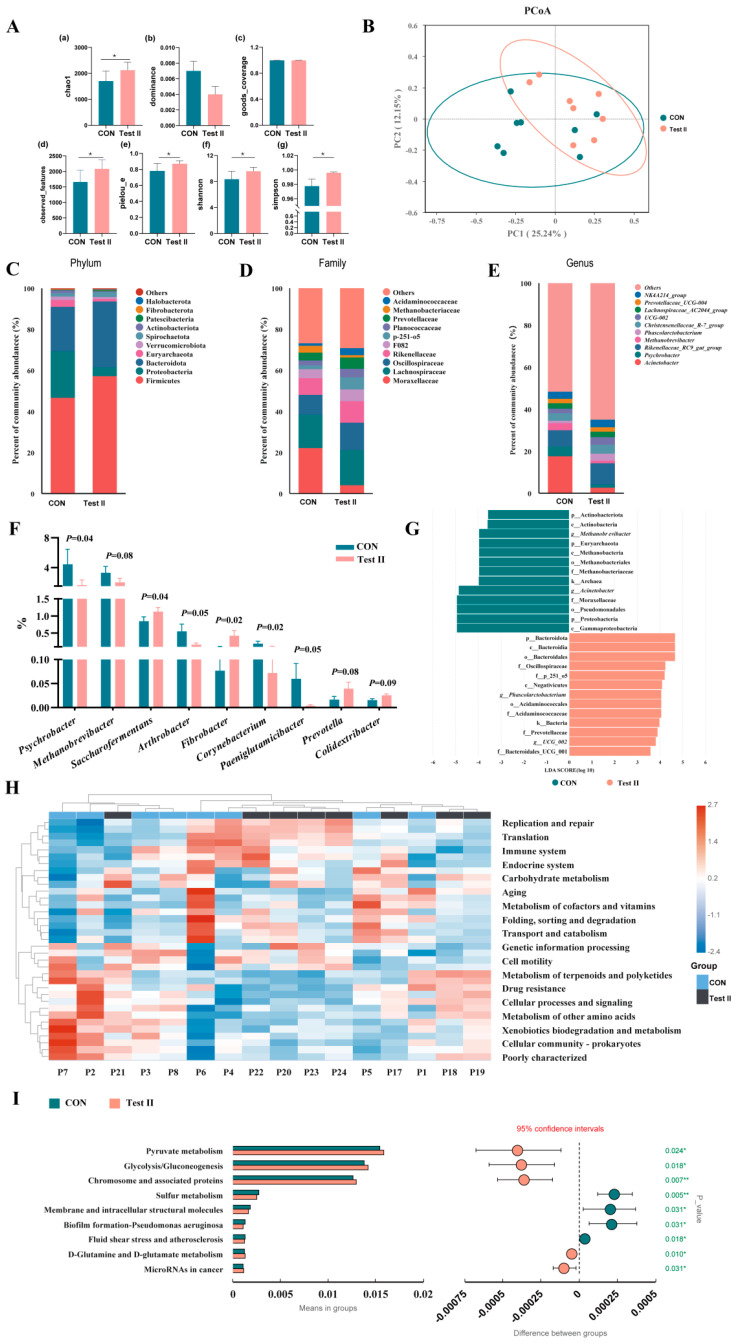
Effects of L-citrulline supplementation on fecal microbiota diversity in pregnant mares. (**A**) Comparison of α-diversity indices. (**a**) chao1 index. (**b**) dominance index. (**c**) good’s coverage. (**d**) observed features. (**e**) Pielou’s evenness index. (**f**) Shannon index. (**g**) Simpson index. (**B**) Principal coordinate analysis (PCoA) plot based on unweighted UniFrac distance, showing β-diversity differences between groups. (**C**–**E**) Relative abundance of microbiota at the phylum, family, and genus levels (top 10). (**F**) Significantly differential taxa at the genus level (identified by rank-sum test with false discovery rate correction, adjusted *p* < 0.05). (**G**) Differential microbial taxa between groups identified by linear discriminant analysis effect size (LEfSe) method (LDA score > 2.0, *p* < 0.05). (**H**) Clustered heatmap of predicted microbial community functional profiles (KEGG Level 2) based on Tax4Fun. (**I**) Microbial functional pathways with significantly different predicted abundances (*p* < 0.05) between CON and Test II groups, identified based on Tax4Fun prediction and T-test (the bar chart on the left shows the mean abundance of each pathway within groups, and the forest plot on the right displays the inter-group difference value and its 95% confidence interval). Significant correlations are marked with asterisks: * *p* < 0.05, ** *p* < 0.01.

To clarify the modulatory effects of L-citrulline supplementation on the gut microbiota, 16S rRNA gene sequencing was performed on fecal samples collected on day 60 of the experiment. Compared to the CON group, supplementation with 30 g/d L-citrulline (Test II group) significantly increased the α-diversity of the fecal microbiota, as evidenced by increases in the Chao1 index, Observed features index, Pielou’s evenness index, Shannon index, and Simpson index (all *p* < 0.05, Wilcoxon rank-sum test, Figure 3A). Principal coordinate analysis (PCoA) based on unweighted UniFrac distance revealed a separation trend in microbial community structure between the two groups (Figure 3B), and the Adonis test confirmed a significant difference in community structure (R^2^ = 0.109, *p* = 0.048). At the phylum level, the dominant phyla in both groups were Firmicutes and Proteobacteria. The Test II group showed a significantly increased relative abundance of Firmicutes and a decreased abundance of Proteobacteria compared to the CON group (Figure 3C). Changes in the relative abundance of dominant bacterial taxa were also noted at the family and genus levels (Figure 3D,E). Linear discriminant analysis effect size (LEfSe) analysis (LDA > 2.0, *p* < 0.05) identified 26 significantly enriched biomarker taxa in the Test II group (Figure 3G), including the family Lachnospiraceae and the order Erysipelotrichales within the phylum Firmicutes, as well as several taxa under the phylum Bacteroidota. At the genus level, a rank-sum test with FDR correction further identified 9 differential bacterial genera (Figure 3F). Among these, the abundances of *Psychrobacter* and *Corynebacterium* were significantly lower, while *Saccharofermentans* and *Fibrobacter* were significantly higher in the Test II group (adjusted *p* < 0.05). Functional prediction of the microbiota based on Tax4Fun (Figure 3H) revealed that the Test II group was enriched in functions related to the “Immune system,” “Endocrine system,” “Translation,” and “Metabolism of cofactors and vitamins,” while the CON group was predominantly enriched for functions such as “Cellular community-prokaryotes” and “Xenobiotics biodegradation and metabolism.” Differential function testing (T-test) indicated that, compared to the CON group, the predicted functional capacity of the microbiota in the Test II group was significantly enhanced in metabolic pathways such as “Pyruvate metabolism” (*p* = 0.024), “Glycolysis/Gluconeogenesis” (*p* = 0.018), and “D-Glutamine and D-glutamate metabolism” (*p* = 0.010), and significantly reduced in pathways like “Membrane and intracellular structural molecules” (*p* = 0.031) and “Biofilm formation-Pseudomonas aeruginosa” (*p* = 0.031) (all *p* < 0.05, Figure 3I).

### 3.8. Association Analysis of Key Physiological Indicators and Intestinal Flora of Mares Under L-Citrulline Regulation

**Figure 4:**
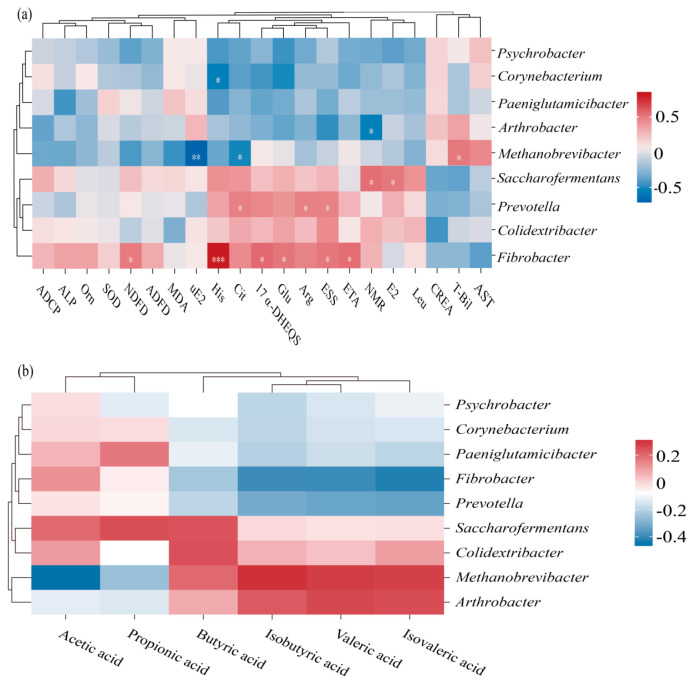
Correlation analysis between key physiological indicators and differential gut bacterial genera in mares. (**a**) Pearson correlation heatmap between key differential physiological indicators and differential bacterial genera. (**b**) Pearson correlation heatmap between fecal short-chain fatty acid concentrations and differential bacterial genera. Each row represents a differential bacterial genus, and each column represents a measured indicator. Red indicates a positive correlation, blue indicates a negative correlation, and the color depth represents the strength of the correlation. Correlation coefficients (r) are indicated by the color scale. Significant correlations are marked with asterisks: * *p* < 0.05, ** *p* < 0.01, *** *p* < 0.001.

To investigate the relationships between host physiology and the gut microbiota under L-citrulline supplementation, indicators showing significant differences from the CON group in apparent digestibility and metabolism, blood biochemistry, antioxidant status, hormones, and amino acid metabolism were selected. Pearson correlation analysis was performed between these indicators and differential microorganisms at the genus level (Figure 4a). The results revealed significant correlations (*p* < 0.05) between several differential bacterial genera and specific physiological indicators. The abundance of *Corynebacterium* was positively correlated with His concentration, while *Arthrobacter* exhibited a significant negative correlation with the nitrogen metabolism rate. *Methanobrevibacter* was positively correlated with T-BiL concentration and negatively correlated with citrulline and urinary E2 concentrations (*p* < 0.01). *Saccharofermentans* showed a positive correlation with plasma E2 concentration and the nitrogen metabolism rate, whereas *Prevotella* was positively correlated with the concentrations of ESS, Arg, and Cit. *Fibrobacter* was positively correlated with the concentrations of ETA, ESS, Glu, 17α-DHEQS, NDFD, and His (*p* < 0.01). Analysis of the associations between six fecal SCFAs and the differential bacterial genera (Figure 4b) showed no significant correlations (*p* > 0.05).

However, the above analysis only indicates statistical associations between the gut microbiota and host physiological/metabolic indicators. The underlying biological mechanisms require further verification through related experiments.

## 4. Discussion

The intestine is the primary site for nutrient digestion and absorption, and its health, closely linked to the microbial community structure, directly impacts the efficiency of nutritional supply to dams in late gestation. This study found that supplementation with 30 g/d of L-citrulline significantly enhanced the apparent digestibility of dietary CP, NDF, and ADF, while also markedly increasing the nitrogen metabolism rate in pregnant mares. These findings align with reports on its metabolic product, arginine. Research indicates that arginine can improve the digestion of dietary protein, energy, and NDF by promoting intestinal absorption, thereby enhancing the overall efficiency of energy and protein utilization [20,21]. Studies in broilers and dairy cows have demonstrated that supplementation with arginine or L-citrulline can upregulate the expression of intestinal nutrient transporters, improve feed conversion ratios, and increase the apparent digestibility of various nutrients [22,23,24]. This study suggests that exogenous L-citrulline, after conversion to arginine in vivo, may enhance the intestinal absorption function of mares through similar mechanisms. Notably, L-citrulline supplementation was found to beneficially reshape the hindgut microbial structure of mares, potentially improving fiber digestion. The hindgut is the primary site for microbial fermentation and fiber degradation in equids, and its microbiota directly influences fiber digestion efficiency [25]. The results indicated that L-citrulline supplementation significantly increased the α-diversity of the fecal microbiota and induced notable changes in the microbial community structure. At the phylum level, the relative abundance of Firmicutes increased, while that of Proteobacteria decreased. Firmicutes contain numerous taxa capable of fermenting complex carbohydrates, and their enrichment is typically linked to enhanced energy harvest and SCFA production [26]. The reduction in Proteobacteria may help lower the load of potential pathogens [27].

Further, significant enrichment of bacterial families involved in fiber degradation and carbohydrate fermentation, such as Lachnospiraceae, Oscillospiraceae, and Prevotellaceae, was observed. Both Lachnospiraceae and Oscillospiraceae are known to be important producers of SCFAs [28]. At the genus level, more detailed microbial structural information revealed that the abundance of fiber-degrading bacteria, *Saccharofermentans* and *Fibrobacter*, also significantly increased. *Saccharofermentans* are involved in carbohydrate degradation and SCFA production, while *Fibrobacter*, a fiber-degrading bacterium, helps improve cellulose digestion and absorption. This is consistent with the increased energy demands during pregnancy [29]. L-citrulline may promote the growth of beneficial bacteria by serving as a nitrogen source [30]. Additionally, interventions that enhance beneficial bacteria have been shown to increase SCFA production, thereby improving host metabolic health [31]. These findings imply that L-citrulline could optimize the intestinal microenvironment by modulating specific bacterial genera, subsequently influencing host metabolism and immune function. While individual fecal VFA concentrations did not reach statistical significance in this study, numerically higher levels of acetate and propionate were observed in the L-citrulline group compared to the CON group, consistent with the enrichment of SCFA-producing bacteria. This study also found that the 30 g/d L-citrulline supplementation enriched functions related to the immune system, endocrine system, translation, and vitamin metabolism. Furthermore, significant enhancements were observed in the functional capacities of energy metabolism pathways such as “Pyruvate metabolism” and “Glycolysis/Gluconeogenesis.” Since these pathways are central to energy production and amino acid metabolism, this suggests that L-citrulline supplementation not only altered the microbiota composition but also optimized the host’s energy utilization and nitrogen metabolism [32].

Various biochemical substances in the blood serve as the material foundation for animal life processes, and their concentrations and fluctuations are significant indicators of animal health. In this study, the 30 g/d L-citrulline supplementation positively influenced several blood biochemical indices in late-pregnant mares. These changes may stem from the impact on liver and kidney function, as well as metabolic balance, following the enhancement of endogenous arginine levels by exogenous L-citrulline. Regarding renal function and protein metabolism, creatinine, the end product of muscle phosphocreatine metabolism, is primarily excreted via glomerular filtration. Its blood concentration is a key indicator of the glomerular filtration rate (GFR), and a decrease suggests enhanced GFR. NO, known for its potent vasodilatory effects, plays a critical role in maintaining renal blood flow and GFR through production by endothelial nitric oxide synthase (eNOS) in the kidneys [33]. Therefore, L-citrulline supplementation may improve renal perfusion by increasing NO bioavailability, thereby accelerating the clearance of metabolic wastes such as creatinine. Although no significant changes were observed in plasma TP and ALB concentrations, the numerically lower values in all groups compared to the control may reflect physiological hemodilution effects due to increased blood volume in late gestation [34]. Regarding liver function and bilirubin metabolism, the plasma T-BiL concentration was significantly lower in the 30 g/d L-citrulline group compared to the control. Bilirubin, a metabolite of heme, is elevated in conditions of increased red blood cell destruction or impaired liver processing capacity. During late gestation, mares often experience heightened oxidative stress and a potential increase in hemolytic risk due to expanded blood volume and active fetal-placental metabolism [35]. Therefore, the significant reduction in plasma T-BiL may be attributed to L-citrulline conversion to arginine in placental tissue, which in turn activates the GSH-Px/SOD pathway, reducing erythrocyte membrane lipid peroxidation and decreasing hemolytic bilirubin production [36]. Additionally, the marked reduction in AST activity in the 30 g/d L-citrulline group suggests a protective effect of L-citrulline on hepatocytes, potentially reducing enzyme release caused by cellular damage. Concerning energy metabolism and osteoblast activity, ALP, a marker enzyme for osteoblasts, showed increased activity, which is typically associated with active placental metabolism and changes in hepatobiliary function [37]. Late gestation is a critical period for fetal skeletal mineralization. The significant increase in ALP activity observed in this study likely reflects enhanced fetal skeletal development, driven by the improved overall metabolic and nutritional status of the dam facilitated by L-citrulline. Meanwhile, the numerically lower plasma Glu concentration in the L-citrulline-supplemented groups compared to the CON group may indicate L-citrulline’s potential to improve insulin sensitivity and promote peripheral glucose utilization [38]. However, this change was not statistically significant and warrants further investigation.

L-citrulline, as a neutral amino acid, primarily undergoes cyclic metabolism in the kidneys. The kidneys absorb L-citrulline from the bloodstream and convert it into arginine via the urea cycle. Research by Jahoor et al. [39] highlights the critical role of the arginine family of amino acids in pregnancy nutrition. The results of this study demonstrate that citrulline supplementation significantly increases the blood concentrations of amino acids involved in the urea cycle in pregnant mares, confirming that exogenous L-citrulline, following efficient absorption, is converted to arginine. This process enhances the flux through the urea cycle [40]. The increased concentrations of urea cycle-related amino acids provide abundant precursors for the synthesis of biologically active molecules critical for pregnancy, such as NO and polyamines, while also boosting the liver’s ammonia detoxification capacity by converting toxic ammonia into non-toxic urea, thereby improving nitrogen metabolic homeostasis. During late gestation, when fetal skeletal muscle and other peripheral tissues experience increased energy demands, amino groups are transferred to pyruvate via transamination to generate alanine. Alanine is then transported via the blood to the liver, where it serves as a key substrate for gluconeogenesis [41]. In this study, plasma alanine concentration was significantly lower in the L-citrulline-supplemented groups, likely because alanine was mobilized and integrated into the glucose-alanine cycle, providing carbon skeletons for the heightened gluconeogenic demand in late gestation. Concurrently, the concentrations of aspartate and glutamate significantly increased. These two amino acids are key nitrogen carriers in the urea cycle and various biosynthetic pathways, suggesting that L-citrulline supplementation enhanced the body’s ammonia detoxification and amino acid metabolic processes. Studies by Neri et al. [42] and van der Linden et al. [43] have shown that the arginine-NO pathway can effectively improve placental blood flow and nutrient delivery efficiency. The changes in amino acid metabolites observed in this study provide strong evidence for the relevance of this pathway in late-pregnant mares.

During late gestation, the rapid fetal growth and increased maternal metabolic demands lead to an elevated production of reactive oxygen species (ROS), making the maintenance of redox balance essential for maternal health and proper fetal development. The endometrium has several potential sources of ROS, with stromal cells primarily generating ROS through the mitochondrial electron transport chain, endoplasmic reticulum, nuclear membrane electron transport, and plasma membrane systems. The high expression of SOD in both endometrial glandular epithelial and stromal cells highlights the important roles of ROS and SOD in regulating uterine function [44]. The observed increases in SOD activity and T-AOC in this study can be attributed to multiple factors. First, L-citrulline is converted to arginine, which is catalyzed by eNOS to produce NO. NO and its metabolites exhibit direct free radical scavenging properties and can enhance the expression and activity of endogenous antioxidants such as SOD and CAT by modulating cellular signaling pathways [45,46]. Additionally, arginine serves as a critical precursor for the synthesis of GSH, the most important non-enzymatic antioxidant within cells, responsible for neutralizing peroxides and hydroxyl radicals, thereby maintaining cellular redox equilibrium [47]. Consequently, L-citrulline supplementation may bolster GSH synthesis and regeneration by increasing arginine availability, synergistically boosting the overall antioxidant defense of cells. The significant reduction in plasma MDA, a stable byproduct of lipid peroxidation, further supports this conclusion. A decrease in MDA levels suggests a reduction in oxidative damage to cellular membranes, which is vital for preserving the integrity of erythrocytes, hepatocytes, and placental trophoblasts [35]. This result also aligns with the observed decrease in plasma T-BiL, reinforcing the enhancement of antioxidant defenses in the mares.

In addition to its positive effects on metabolism and the antioxidant system, this study also revealed that L-citrulline supplementation significantly influenced the reproductive endocrine status of mares in late gestation. This was evident from the increased plasma concentrations of E2 and P4, as well as elevated levels of conjugated estrogens in the urine. These findings suggest that L-citrulline supplementation may positively regulate hormone synthesis and metabolic pathways critical for maintaining pregnancy. The balance of steroid hormones such as E2 and P4 is essential for embryonic development and pregnancy maintenance in mares [48]. As a precursor for NO synthesis, L-citrulline is known for improving vascular endothelial function and enhancing tissue blood perfusion [14]. Adequate placental blood flow not only facilitates the delivery of nutrients and oxygen to the fetus but may also optimize the functional state and hormone-synthesizing capacity of placental trophoblast cells. Moreover, L-citrulline and its metabolites contribute to polyamine biosynthesis, which plays a pivotal role in regulating cell proliferation, differentiation, and gene expression—processes that are essential for placental development, maturation, and endocrine function [49]. Thus, L-citrulline supplementation may support the healthy development and function of the placenta, fostering an environment conducive to steroid hormone synthesis. The observed increase in urinary conjugated estrogen levels further supports the notion of enhanced estrogen metabolic flux [50,51]. After exerting their biological effects, estrogens are primarily conjugated in the liver through sulfation, rendering them water-soluble for excretion in the urine. Elevated levels of conjugated estrogens indicate an accelerated rate of estrogen synthesis, metabolism, and excretion. These findings suggest that L-citrulline supplementation may enhance the urea cycle, improve nitrogen metabolism and nutritional status, and promote uterine and placental blood perfusion. Additionally, L-citrulline may regulate the activity of steroidogenic enzymes through cell signaling pathways [52], thereby enhancing estrogen biosynthesis and its subsequent conjugation in the liver, which results in higher concentrations of specific sulfate conjugates in the urine [51].

However, the study has certain limitations. Despite the sample size being consistent with standard practice in the field, it remains relatively small, which may limit the ability to detect more subtle changes in health indicators. Additionally, as the study was conducted with a specific breed and physiological stage, the long-term effects on postpartum mares and their offspring require further investigation. The mechanistic explanations for the observed physiological changes are largely inferred from correlations with health indicators. Future research should validate the specific molecular pathways through which L-citrulline influences placental function, immune regulation, and gut microbiota through more detailed multi-omics analyses and targeted experiments.

## 5. Conclusions

This study demonstrates that dietary supplementation with L-citrulline offers a promising nutritional strategy for enhancing the health of Yili mares in late gestation. Through a comprehensive assessment encompassing nutrient digestibility, metabolism, blood parameters, antioxidant function, hormone levels, amino acid metabolism, and gut microbiota, a daily dose of 30 g of L-citrulline per mare yielded the most favorable health outcomes under the conditions of this study. Specifically, this dosage significantly improved CP digestibility, nitrogen metabolism, and plasma antioxidant capacity, while also elevating estradiol and urinary conjugated estrogen levels. Furthermore, it promoted the synthesis of key urea cycle amino acids and increased gut microbiota diversity, particularly enhancing populations of beneficial bacteria such as Lachnospiraceae. In conclusion, supplementation with 30 g/d of L-citrulline holds significant potential for improving nutritional metabolism, reducing oxidative stress, balancing endocrine function, and optimizing gut microbiota in late-pregnant mares. Further studies are needed to explore the underlying mechanisms and assess the long-term benefits and applicability of this nutritional approach across diverse equine populations.

## Figures and Tables

**Table 1:** Diet Composition and Nutritional Levels (Dry Matter Basis).

Feed Composition	Content %	Nutritional Level ^(2)^	Content
Silage	30.00	DM (%)	73.65
Alfalfa straw	18.00	OM (%)	87.25
Oat straw	15.00	GE (MJ/kg)	15.86
Wheat straw	17.00	CP (%)	8.34
Corn	4.50	EE (%)	1.84
Oat	6.50	NDF (%)	45.89
Wheat	8.00	ADF (%)	27.10
Salt	0.10	Ca (%)	0.44
Premix ^(1)^	0.90	P (%)	0.27
In total	100.00		

^(1)^ Note: The premix provided the following per kg of the concentrate supplement: Vitamin A 6500 IU, Vitamin D_3_ 1400 IU, Vitamin B_1_ 21.26 mg, Vitamin B_2_ 333.20 mg, Vitamin B_6_ 1.20 mg, Vitamin E 850 mg, Biotin 5 mg, Pantothenic acid 20.46 mg, Nicotinamide 84.85 mg, Cu (as copper sulfate) 35.00 mg, Fe (as ferrous sulfate) 127.62 mg, Mn (as manganese sulfate) 133.54 mg, Zn (as zinc sulfate) 150.00 mg, I (as potassium iodide) 21.46 mg, Se (as sodium selenite) 51.93 mg, Co (as cobalt chloride) 4.11 mg. ^(2)^: Nutritional values are based on laboratory analysis.

**Table 6:** Effects of Supplementing L-citrulline on the pH and VFA of feces in pregnant mares.

Items	CON	Test II	*p*-Value
pH	6.64 ± 0.22	6.51 ± 0.32	0.38
Acetic acid (µg/g)	522.08 ± 70.65	578.45 ± 165.17	0.07
Propionic acid (µg/g)	132.16 ± 26.45	136.91 ± 41.4	0.19
Butyric acid (µg/g)	39.57 ± 12.32	31.04 ± 19.17	0.23
Isobutyric acid (µg/g)	53.88 ± 22.86	61.28 ± 20.37	0.80
Valeric acid (µg/g)	50.18 ± 10.52	46.26 ± 11.34	0.93
Isovaleric acid (µg/g)	37.37 ± 5.92	34.49 ± 5.80	0.98
TVFA (µg/g)	835.22 ± 101.69	888.43 ± 221.31	0.10

## Data Availability

The original contributions presented in this study are included in the article. Further inquiries can be directed to the corresponding author.

## Abbreviations

The following abbreviations are used in this manuscript

DM	Dry Matter
OM	Organic Matter
DE	Digestible Energy
CP	Crude Protein
EE	Ether Extract
NDF	Neutral Detergent Fiber
ADF	Acid Detergent Fiber
Ca	Calcium
P	phosphorus
N	Nitrogen
TP	Total Protein
ALB	Albumin
GLB	Globulin
UREA	Urea
CREA	Creatinine
TC	Total Cholesterol
T-BiL	Total Bilirubin
Glu	Glucose
ALT	Alanine Aminotransferase
AST	Aspartate Aminotransferase
ALP	Alkaline Phosphatase
γ-GT	γ-Glutamyl Transferase
CK	Creatine Kinase
LDH	Lactate Dehydrogenase
Mg	Magnesium
T-AOC	Total Antioxidant Capacity
SOD	Superoxide Dismutase
GSH-Px	Glutathione Peroxidase
CAT	Catalase
MDA	Malondialdehyde
GnRH	Gonadotropin-Releasing Hormone
LH	Luteinizing Hormone
FSH	Follicle-Stimulating Hormone
E2	Estradiol
P4	Progesterone
T	Testosterone
E1	Estrone
ESS	Estrone Sulfate
EQS	Equilin Sulfate
17α-DHEQS	17α-Dihydroequilin Sulfate
